# KLF4和SPARC在非小细胞肺癌中的表达及其相关性研究

**DOI:** 10.3779/j.issn.1009-3419.2012.12.05

**Published:** 2012-12-20

**Authors:** 志平 张, 洲 王, 相燕 刘, 墨 史, 钢 陈, 波 张, 哲 李, 亮 宋

**Affiliations:** 1 250021 济南，山东大学附属省立医院胸外科 Department of Thoracic Surgery, Provincial Hospital Affiliated to Shandong University, Jinan 250021, China; 2 250013 济南，山东大学附属济南市中心医院胸外科 Department of Thoracic Surgery, Jinan Central Hospital, Jinan 250013, China; 3 250013 济南，山东大学附属济南市中心医院病理科 Department of Pathology, Jinan Central Hospital, Jinan 250013, China

**Keywords:** 肺肿瘤, KLF4, SPARC, Lung neoplasms, Krüppel-like factor 4, Secreted protein acidic and rich in cysteine

## Abstract

**背景与目的:**

已有研究证实*KLF4*基因（Krüppel-like factor 4）和富含半胱氨酸的酸性分泌蛋白（secreted protein acidic and rich in cysteine, SPARC）与肿瘤的发生发展密切相关。本研究旨在检测KLF4和SPARC蛋白在非小细胞肺癌（non-small cell lung cancer, NSCLC）中的表达，并结合临床病理特征来探讨KLF4和SPARC的临床意义及相关性。

**方法:**

应用免疫组织化学方法检测89例NSCLC组织及正常肺组织中KLF4和SPARC的表达。

**结果:**

KLF4在癌旁正常肺组织阳性表达率为88.8%，NSCLC组织为42.7%（*P* < 0.05）；有、无淋巴结转移者的KLF4阳性表达率分别为31.3%和56.1%（*P* < 0.05）；KLF4的表达与肿瘤临床分期有关（*P* < 0.05），随着临床分期等级的增加，KLF4表达呈现递减趋势。SPARC在NSCLC组织的阳性表达率为70.8%，癌旁正常肺组织为7.9%（*P* < 0.05）；低、高分化癌的SPARC阳性表达率无统计学差异（*P* > 0.05）；有、无淋巴结转移者的SPARC阳性表达率分别为81.3%和58.5%（*P* < 0.05）；其表达与肿瘤的临床分期相关（*P* < 0.05）。KLF4和SPARC的表达均与患者的性别、年龄和肿瘤大小无关（*P* > 0.05）。SPARC和KLF4在NSCLC中的表达呈负相关（*r*=-0.245, *P* < 0.05）。

**结论:**

KLF4低表达及SPARC的过表达与NSCLC的发生及其生物学行为密切相关，可能作为NSCLC诊断及分期预后的指标。

## Abstract (en)

**Background and objective:**

It has been proven that the development and biological behavior of lung carcinoma is affected by a number of signaling pathway elements. The expression levels of Krüppel-like factor 4 (KLF4) and secreted protein acidic and rich in cysteine (SPARC) were correlated with tumorigenesis and prognosis. This study aims to investigate KLF4 and SPARC expression and their correlation with the clinical characteristics of non-small cell lung cancer (NSCLC).

**Methods:**

KLF4 and SPARC expression was examined immunohistochemically in NSCLC and normal lung tissues from 89 patients.

**Results:**

SPARC expression was increased in the tumor specimens compared with the control tissue (70.8% *vs* 7.9%, *P* < 0.05), whereas KLF4 protein was reduced compared with that in the control tissue (42.7% *vs* 88.8%, *P* < 0.05). KLF4 expression was significantly correlated with lymph node metastasis and clinical stage (*P* < 0.05). KLF4 expression in NSCLC decreased with the increasing clinical stage. The positive rate of SPARC expression in NSCLC with lymph node metastasis was significantly higher than that without lymph node metastasis (81.3% *vs* 58.5%, *P* < 0.05). Lung carcinomas in stages Ⅰ and Ⅱ disease had significantly lower rates of positive SPARC expression than that in stages Ⅲ and Ⅳ diseases (*P* < 0.05). Both were not related to age, sex, and tumor size (*P* > 0.05). KLF4 expression was negatively correlated with SPARC expression in NSCLC (*r*=-0.245, *P* < 0.05).

**Conclusion:**

The SPARC overexpression may play an important role in the initiation and development of NSCLC, whereas KLF4 inhibits this process. These proteins may be used as marker for evaluating the biological characteristics and clinical stages of NSCLC.

肺癌的发病率及死亡率逐年上升，目前己成为世界上死亡率第一位的恶性肿瘤^[1]^。肺癌的5年生存率在美国仅为15%，我国则更低。肺癌的发生、发展是一个多因素、多步骤的复杂过程，常与原癌基因激活、抑癌基因下调失活以及侵袭、转移等因素相关。*KLF4*基因（Krüppel-like factor 4）是一种在人类多种组织中广泛表达的锌指转录因子，在许多不同的生理活动包括生长、分化和正常组织稳态的维持中具有重要作用^[2]^。近期研究^[3]^表明KLF4在肿瘤的发生与发展中具有重要的作用。而KLF4在肺癌中的表达及其对肺癌发生发展的关系尚不清楚，与肺癌相关性的报道比较少见。富含半胱氨酸的酸性蛋白（secreted protein acidic and rich in cysteine, SPARC）与某些细胞外基质成份、生长因子和细胞因子作用参与组织重建、形态生成、细胞迁移和增殖，调节机体的多种生理和病理过程，且与某些肿瘤的临床分期和预后关系密切^[4]^，但SPARC在肺癌中的作用仍不明确。同时，有关KLF4、SPARC在肺癌中表达关系的研究较少报道。本研究采用免疫组化法检测非小细胞肺癌（non-small cell lung cancer, NSCLC）组织中KLF4、SPARC的表达情况，以探讨其与NSCLC发生、发展的关系，为NSCLC的诊断和预后评价寻求客观指标。

## 1 资料和方法

### 1.1 病例选择

随机选取山东省立医院胸外科2009年8月-2010年8月收治的NSCLC病例89例，其中男53例，女36例；年龄33岁-84岁，平均56岁，均经术后病理检测证实。术前均未接受放疗和化疗，临床资料完整。另选择相应距肿瘤边缘5 cm以外的正常肺组织作为对照。

### 1.2 主要试剂

KLF4、SPARC兔抗人多克隆抗体及免疫组化SP试剂盒均购自福州迈新生物技术公司。抗体克隆号：KLF4 ab34814，SPARC sc13314；抗体稀释浓度为1:100。

### 1.3 检测方法

2名病理学专家对HE染色的病理切片进行筛选，找出肿瘤组织中有代表性的区域，并在相应石蜡块上作标记。应用免疫组化SP法检测标本中的KLF4与SPARC蛋白。严格按试剂盒说明书进行。以PBS代替一抗作为阴性对照，用已知阳性切片作阳性对照，光镜观察。

### 1.4 结果判定

由2位有经验的病理专家对结果进行评定。KLF4蛋白在石蜡切片中阳性染色主要定位于细胞核，少数可见于细胞浆，呈现棕黄色颗粒。根据肿瘤细胞显色的比例及染色强度对KLF4表达做半定量判定^[5]^。按阳性细胞率评分：阳性细胞数占11%-50%为2分；51%-80%为3分； > 80%为4分。按显色程度评分：染色弱为1分；中等染色为2分；强染色为3分。将两种评分结合起来分为3级：无论染色强度如何，细胞阳性率 < 10%为阴性（-）；3分-5分为弱阳性（+）；6分-7分为强阳性（+ +）。

SPARC蛋白主要表达于细胞浆，少数可同时表达于细胞核，参照文献的判断标准^[6]^，根据着色强度及阳性细胞百分比进行评分：无色为0分，淡黄色（弱阳性）为1分，棕黄色（中等染色强度）为2分，棕褐色（强阳性）为3分；阳性细胞 < 1%为0分，1%-24%为1分，25%-49%为2分，50%-74%为3分，75%-100%为4分。免疫反应得分为百分率得分与免疫染色强度得分的乘积。

### 1.5 统计学方法

采用SPSS 13.0统计分析软件，率的比较采用*χ*^2^检验及*Fisher*精确概率法，相关性分析采用*Spearman*等级相关，*P* < 0.05为差异有统计学意义。

## 2 结果

### 2.1 KLF4和SPARC蛋白在NSCLC组织中的表达

KLF4染色主要位于细胞核，部分位于细胞浆，呈现棕黄色颗粒。KLF4蛋白在正常肺组织标本中可见KLF4蛋白强阳性表达，阳性表达率为88.8%，而KLF4在NSCLC组织中表达率为42.7%（*P* < 0.05）（图 1A）。

**1:**
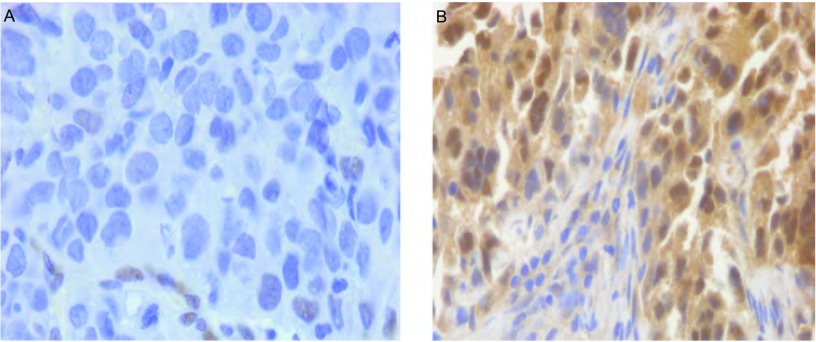
KLF4、SPARC蛋白在非小细胞肺癌组织中的表达（SP, ×400）。A：KLF4在肺癌组织中弱阳性表达；B：SPARC在肺癌组织中阳性表达。 KLF4 and SPARC expression in non-small cell lung cancer (NSCLC) (SP, ×400). A: Weak positive expression of KLF4 in NSCLC; B: Positive expression of SPARC in NSCLC.

SPARC主要表达于胞浆，少数也可见于胞核，其在NSCLC中的阳性表达率为70.8%，与正常肺组织（7.9%）相比，差异具有统计学意义（*P* < 0.05）（图 1B）。

### 2.2 KLF4、SPARC阳性表达与NSCLC临床病理特征的关系

见表 1。KLF4在NSCLC组织中的表达与患者淋巴结转移及临床分期有关，有、无淋巴结转移者的KLF4阳性表达率分别为31.3%和56.1%（*P*=0.03），随着临床分期等级的增加，KLF4表达呈现递减趋势（*P*=0.03）；KLF4表达与患者的性别、年龄及肿瘤细胞分化程度等无关（*P* > 0.05）。SPARC在NSCLC组织中的表达与患者淋巴结转移及临床分期有关，有、无淋巴结转移者的阳性表达率分别为81.3%和58.5%（*P*=0.02），随着临床分期等级的增加，SPARC表达呈现递增趋势（*P*=0.04）；SPARC表达与患者的性别、年龄及肿瘤细胞分化程度等无关（*P* > 0.05）。

**1:** KLF4、SPARC阳性表达与非小细胞肺癌临床病理特征的关系 Relationship between KLF4 and SPARC expression and clinicopathological characteristics in NSCLC

Variable/Category	*n*	KLF4 protein		*P*	SPARC		*P*
		+	-		+	-	
Gender				0.52			0.49
Male	53	21	32		39	14	
Female	36	17	19		24	12	
Age (years)				0.50			0.62
≥60	59	27	32		43	16	
< 60	30	11	19		20	10	
Type				0.34			0.14
SqCC	42	20	22		32	10	
AdC	38	13	25		23	15	
MX	9	5	4		8	1	
Size (cm)				0.50			0.11
≤3	34	17	17		20	14	
3-5	40	16	24		30	10	
> 5	15	5	10		13	2	
Differentiation				0.39			0.94
WD	12	5	7		8	4	
MD	41	18	23		29	12	
PD	36	13	23		26	10	
TNM				0.03			0.04
Ⅰ	12	8	4		5	7	
Ⅱ	28	16	12		18	10	
Ⅲ	42	12	30		34	8	
Ⅳ	7	2	5		6	1	
Lymphaden				0.03			0.02
N_1-3_	48	15	33		39	9	
N_0_	41	23	18		24	17	
SqCC: squamous cell carcinoma; AdC: adenocarcinoma; MX: mixed carcinoma; WD: well differentiation; MD: moderately differentiation; PD: poorly differentiation.							

### 2.3 KLF4与SPARC表达的关系

KLF4和SPARC共同表达阳性者为22例（24.7%），共同表达阴性者为10例（11.2%）；KLF4表达阳性，SPARC表达阴性者16例（18.0%）；KLF4表达阴性，SPARC表达阳性者41例（46.1%），两者的表达呈负相关（*r*=-0.245, *P* < 0.05）。

## 3 讨论

*KLF4*基因是一种真核生物锌指蛋白转录因子，通过激活或者抑制目的基因的转录来调控基因表达的时间特异性和组织特异性，在细胞生长、分化及凋亡等过程中发挥重要作用^[7]^。KLF4在肿瘤中的表达具有类型特异性。在人FAP患者的结肠腺瘤中KLF4表达下调，在多发性结肠腺瘤及结肠癌KLF4的表达也明显下调^[8]^。但是，KLF4却在口腔上皮异常增生，在口腔癌以及乳腺癌的全层上皮过度弥漫表达^[9]^。目前对于KLF4表达的类型特异性的具体机制尚不明确，有待进一步研究。Chen等^[10]^研究显示，KLF4通过调节下游靶基因的转录表达而在细胞的周期调控、增生分化与程序性死亡中具有重要作用。KLF4可激活p21、角蛋白4、角蛋白19和EBVE-L2等与细胞分化相关的基因的启动子，而对细胞色素P450-CYPlA1、细胞周期蛋白D1、β2球蛋白等促进细胞周期循环的基因有抑制作用。Yoon等^[11]^研究发现，KLF4能通过与细胞周期蛋白Bl启动子中富含GC的元件结合而抑制其转录活性，从而在维持G_2_/M细胞周期检查点的完整性中担任重要角色。作为一个新发现的转录因子，KLF4通过调节其下游目标基因的转录表达而影响细胞整体的生理功能。KLF4在细胞的增生和分化中担任重要角色，提示它与肿瘤发生发展有密切联系。Wei等^[5]^证明KLF4在肿瘤发生期的胃肠道上皮表达下调，通过调节一些细胞周期调控和分化相关基因的表达而在细胞增殖分化和细胞周期调控中承担重要角色，并与胃癌的发生发展及预后密切相关。近来，Zhao等^[12]^采用半定量RT-PCR技术检测出，KLF4 mRNA的表达与结肠癌的病理分期和病理分级负相关，提示KLF4的表达与该肿瘤的侵袭转移和预后有关，可能参与了结肠癌的发生或发展过程。体外实验则证明KLF4过表达可抑制结肠肿瘤的形成、转移和浸润。恢复KLF4表达后肿瘤转移受到抑制，细胞周期停滞，癌细胞发生凋亡。可见，KLF4表达与胃癌、结肠癌密切相关，可作为判断疾病不良预后的指标。本研究的结果显示KLF4在正常肺组织中的表达明显增强，而在NSCLC组织中表达明显下调，且与NSCLC的分化程度、临床分期及淋巴结转移关系密切，由此可见KLF4表达下调在NSCLC的发生与发展中可能发挥重要的作用。

SPARC由纤维原细胞、成骨细胞、软骨细胞、上皮细胞和血小板等产生。SPARC可与某些细胞外基质成份、生长因子和细胞因子作用，参与组织重建、形态生成、细胞迁移和增殖，调节机体的多种生理和病理过程^[13]^。SPARC在多种恶性肿瘤被确认为表达上调, 如结、直肠癌等^[14]^，但也有些肿瘤组织SPARC呈低水平表达或不表达，如卵巢癌等^[15]^，具体机制尚待进一步研究。在NSCLC中肿瘤细胞和肿瘤基质细胞都可以合成SPARC^[16]^，是否会对NSCLC的侵袭和转移产生不同的影响尚不明了。SPARC表达于肿瘤中的新生血管内皮细胞而不表达于成熟血管的内皮细胞，提示SPARC与肿瘤新生血管的生成有关。SPARC与多种肿瘤的发生、发展、侵袭和转移相关，且与某些肿瘤的临床分期和预后关系密切，但SPARC在肺癌中的作用仍不明确，研究结果也不尽相同。本文结果显示SPARC在NSCLC组织中表达上调，在正常肺组织中不表达，而且SPARC蛋白的表达强度与NSCLC的淋巴结转移相关，提示SPARC基因是一种NSCLC相关肿瘤基因，SPARC蛋白高表达的NSCLC有更强的侵袭和转移能力，SPARC有可能成为判断或预测NSCLC淋巴转移的标志物。SPARC高表达的患者临床分期偏晚，预后不良，SPARC也可能是一种NSCLC患者预后判断或疗效检测的标志物。因本文随访时间较短，SPARC表达与预后的关系还需进一步深入研究。

本组研究中显示KLF4和SPARC基因在NSCLC组织中的表达呈负相关（*P* < 0.05），两者可能协同参与了NSCLC的发生发展。Zhou等^[17]^发现，KLF4可抑制NSCLC中SPARC mRNA与蛋白的表达，从而降低肺癌细胞侵袭能力，可见KLF4、SPARC表达及相互作用与NSCLC密切相关，但其作用机制尚不清楚，有待进一步深入研究。本实验中KLF4失表达的NSCLC中SPARC表达全部为阳性，提示NSCLC组织中*KLF4*基因失活可能促使*SPARC*基因表达增加，导致肿瘤发生发展及转移。因此同时检测两者的表达有助于进一步了解NSCLC的生物学行为，并为判断NSCLC的淋巴转移、预后评估提供有价值的参考指标。但是肿瘤的发生发展是多因素、多基因共同作用的结果，在未来NSCLC的治疗中分子靶向药物及多基因联合治疗是研究的趋势，可能成为NSCLC靶向治疗的新策略。这些发现为进一步探讨NSCLC中*KLF4*、*SPARC*基因表达关系及作用机制提供了线索，值得进一步探讨研究。
